# Supplementary material for: Are carbon emissions trading and green financial instruments synergistic? -Comprehensive quantitative research based on content analysis
Source: PLoS One. 2024 Mar 7;19(3):e0298601. doi: 10.1371/journal.pone.0298601 (PMC10919601; doi:10.1371/journal.pone.0298601)
Supplement: S1 Data — (ZIP) [file pone.0298601.s001.zip › Data/地级市Z_F工作报告.zip!/║■▒▒/╛ú╓▌╩╨/└┤╫╘╣1⁄2╓┌║┼í╛┬φ┐╦╩2╛▌═°í┐_2011.docx]

各位代表：

现在，我代表市人民政府，向大会作工作报告，请与《荆州市经济和社会发展第十二个五年规划纲要（草案）》一并审议，并请市政协委员和其他列席人员提出意见。

一、过去六年的工作回顾

本届政府任期的六年，是宏观经济形势复杂多变、自然灾害频繁发生、机遇挑战相互交织的六年，也是建市以来经济社会发展最快、城乡面貌变化最大、人民群众受惠最多的六年。在省委、省政府和市委的正确领导下，在市人大、市政协的监督支持下，市政府深入贯彻落实科学发展观，团结和带领广大干部群众，开拓进取、克难奋进，有效化解国际金融危机对经济的不利影响，积极抗御低温冰雪、外洪内涝等严重自然灾害，成功举办奥运圣火传递、第二届全球华商500强高科技论坛、中国荆州淡水渔业展示交易会等重大活动，全面超额完成“十一五”规划目标任务。

--六年来，经济发展明显加速，综合实力迈上新台阶。我们牢牢把握荆州发展的阶段性特征，着力实施“工业兴市”战略，在加快发展中调结构、转方式、促转型，实现了从走出低谷、奋力爬坡到步入快车道的跨越。

经济规模迅速扩大。2010年末，地区生产总值837.1亿元、“十一五”年均增长12.2%，为“十五”末的2倍，三次产业结构调整到27.6：38.9：33.5，第二产业比重比“十五”末提高7.3个百分点；全社会固定资产投资600.9亿元、年均增长40%，为“十五”末的5.1倍；社会消费品零售总额471.1亿元、年均增长18.9%，为“十五”末的2.1倍；财政收入73.7亿元、年均增长24.7%，为“十五”末的3倍，其中地方一般预算收入27.6亿元、年均增长18%，为“十五”末的2.3倍。

工业主导作用增强。2010年末，规模以上企业达到1225家、净增657家；规模以上工业增加值264.4亿元、“十一五”年均增长24%，为“十五”末的3.4倍；产销规模过30亿元的产业集群6个，列入省重点扶持的产业集群8个，年销售收入过亿元的企业137家、过10亿元的企业11家；精品名牌实现零的突破，中国名牌、中国驰名商标分别达到6个和4个。县域经济实力增强，荆州区率先跻身全省县域综合实力20强。

现代农业加快发展。养殖业比重超过种植业，占农业总产值的51.5%。粮棉油、畜禽水产、平原林业等优势农业板块不断壮大，粮食总产突破75亿斤，油菜、棉花、水产品产量居全省首位，荣获“中国淡水渔业第一市”称号。农业产业化水平稳步提高，监利、洪湖农产品加工园被确定为省市县共建示范园区，福娃和洪湖鱼米乡进入全省农产品加工业前十强；省级以上龙头企业达到55家，居全省之首。水利建设得到加强，累计投入资金47亿元，完成长江干堤等堤防加固900公里，水库除险22座，更新改造泵站19处61座，恢复和改善灌溉面积150万亩。

消费市场繁荣活跃。成功引进沃尔玛、大润发等一批大型商贸企业，建成北京路地下商城，中心城区核心商圈基本形成。加快推进“万村千乡”、“双百”市场工程，新建改造农家店3443家，国家级商品市场达到3家，两湖农产品交易物流中心贸易规模超过100亿元。文化旅游加快发展，中国大遗址保护荆州片区建设正式启动，荆州古城、“洪湖岸边是家乡”成为鄂西圈重点建设的十大核心景区，接待游客达到912.6万人次，旅游收入达到51.7亿元。金融业发展出现重大转机，2007年开始全面盈利，金融机构存款余额突破千亿元大关、达到1036.1亿元，贷款余额达到461.1亿元，我市连续四年被评为省“A级信用市州”，重塑了荆州良好的信用形象。

--六年来，基础设施明显改善，城乡面貌发生新变化。我们坚持扩内需、强支撑，以大投入促进大建设，以大建设改善硬环境，发展条件稳步改善，区位优势逐步显现。

交通条件大为改善。累计完成投资132亿元，随岳高速、荆东高速、荆岳长江大桥、东荆河大桥、盐卡港二期等工程建成运行，荆州人民期盼多年的汉宜高铁荆州段、荆沙铁路改建、洪监高速、江南高速动工建设，荆岳铁路、城市大外环开工在即，建成农村公路13700公里，所有乡镇通达三级以上公路，所有行政村通达油路或水泥路。

城市形象大幅提升。城市规划体系逐步完善，新一轮城市总规和工业新区、海子湖生态文化旅游区等片区规划编制完成。中心城区累计投入24.3亿元，荆沙大道全线贯通，北京路、荆中路等30条主次干道黑化改造升级，荆沙河、护城河、内荆襄河清淤整治和水系连通工程完成，中心城区3座污水处理厂建成营运，沿江、沿河、沿湖绿化建设步伐加快，城市污水处理率达到80%，实现省级卫生城市创建目标。松滋、石首被评为省级文明城市，洪湖被评为省级卫生城市和园林城市。全国重点镇和历史文化名镇分别达到7个和3个，省级重点镇达到10个，预计全市城镇化率达到45%，比“十五”末提高10.4个百分点。

新农村建设大力推进。强农惠农政策得到落实，累计发放各类惠农补贴31亿元，取消农业税、农业特产税和牲畜屠宰税，农民负担下降到人平33.1元、亩平23.1元。实施高产农田土地整治项目86个，建设规模208万亩，新增耕地6.5万亩。建成户用沼气池38.5万口，新增农村安全饮水人口175万人。新农村建设试点乡镇达到43个，覆盖55%的农村人口，仙洪试验区荆州片、花基台、李埠、孟溪大垸等成为新亮点，全市农村呈现经济发展提速、设施条件改善、村容村貌明显改观的大变化。

--六年来，改革开放明显加快，发展活力得到新提升。我们坚持以开放促改革，以深化改革突破体制机制障碍，开放型经济格局初步形成。

体制机制改革不断深化。国有工业企业改革基本完成，国有资产监管营运体系基本建立，成功化解天发集团金融风险，天颐科技恢复上市。农村综合配套改革纵深推进，“以钱养事”新机制进一步完善，集体林权改革、国有农场改革任务基本完成。市县两级政府机构改革完成，事业单位分类改革稳步推进，行政审批制度“三集中、三到位”改革和中心城区城管体制改革取得实效。教育、卫生等事业单位绩效工资改革启动，国家基本药物制度和公立医院改革试点顺利开展。财政管理体制改革不断深入，实现“乡财县管”和农村教师工资县级统一发放、账户直达，收支两条线、国库集中支付、政府采购、部门预算和公务卡结算方式全面实施。土地收储制度建立并逐步完善，招投标“五统一”综合监管模式在全国推广。

对外开放水平不断提高。开放平台进一步扩大，全市开发区新增2个，达到10个；荆州开发区园区平台初具规模，产业集聚和开放竞争优势明显增强。招商引资成效显著，2010年实际到位资金258亿元、年均增长40%以上，是“十五”末的5倍，利洁时、中化、中粮、中纺、中房、美的、三安等一批世界500强、央企和上市公司入驻荆州。外贸出口达到5.74亿美元，是“十五”末的2.4倍。

--六年来，社会事业明显进步，人民生活达到新水平。我们坚持以人为本，将关注民生、维护民利、排解民忧作为政府工作的出发点和落脚点，努力让全市人民共享改革发展成果。

人民生活稳步改善。城市居民可支配收入达到14708元、“十一五”年均增长12.7%，农民人均纯收入达到6453元、年均增长15.8%。国家创业型城市创建活动扎实推进，累计发放小额担保贷款4.9亿元，新增城镇就业53.9万人，城镇登记失业率控制在4.2%以内，“百姓创家业”经验在全国推广。社会保险参保人数达到369万人，社保收入35.8亿元、年均增长29.3%。连续六年提高养老金发放标准，企业离退休职工养老金月平达到1010.8元。城镇医保、农民合作医疗参保人数分别达到204万人和405万人，率先在全省实现门诊统筹全覆盖。城乡低保覆盖面达到14.2万人和17.9万人，月人均补助水平分别达到160元和60元。保障性住房建设得到加强，累计投入廉租房建设资金3.5亿元，发放住房租赁补贴3.6万户、1.03亿元，建成经济适用房2630套。累计归集住房公积金31.2亿元，发放公积金贷款14.4亿元。累计投入扶贫开发资金3.74亿元，老区贫困地区生产生活条件不断改善。

社会事业协调发展。企业自主创新能力不断增强，高新技术企业达到34家，获得省以上科技成果进步奖142项。“两基”教育通过国家评估验收，义务教育经费保障新机制建立，化解“普九”债务3.54亿元。职业教育和高等教育稳步发展，在校学生分别达到10万人和12万人。文化文博、广播影视、社会科学、文学艺术、新闻出版、体育事业进一步繁荣，乡镇综合文化站、农家书屋等惠民工程建设加快，荆州博物馆获评国家一级博物馆，《垄上行》荣获“全国十大最具原创精神电视栏目”称号，成功举办市四运会、关公刀会和龙舟赛。公共卫生服务体系不断完善，建成市惠民医院、6个县市惠民医疗窗口和936个农村标准化卫生室，社区卫生服务机构合格率达到100%。血防整县推进和四湖流域综合治理成效明显，完成省下达的“以机代牛”任务，血吸虫病疫情控制达标通过国家验收。社区基础设施建设得到加强，中心城区累计投入资金3020万元，沙市区荣获“全国和谐社区示范城区”称号。第六次人口普查工作取得阶段性成果。人防工程建设取得重大进展，荣获“全国人民防空先进城市”称号；国防动员、国防教育和民兵预备役、“双拥”等工作扎实推进，石首市荣获“全国双拥模范城”称号。编制、统计、物价、供销、盐务、邮政、移民、食品药品监管、民族宗教、外事侨务、台湾事务、气象、地震、档案、史志、保密和无线电等工作都有新的进步，妇女儿童、老龄、残疾人等事业进一步发展。

基本国策得到落实。努力维持低生育水平，计划生育工作连续五年受到省委、省政府表彰。严守耕地红线，保护经验在全国推广。强化生态环境保护，狠抓节能减排与污染治理，依法关停小造纸、小印染、小水泥、小炼钢企业61家，完成减排项目183个，化学需氧量和二氧化硫排放分别下降15%和8%，单位生产总值能耗下降20.6%。四湖流域等重点区域生态修复和城镇生活污水治理步伐加快，完成洪湖拆围工作，建成城镇污水处理厂21个，生态环境进一步好转。

--六年来，政府自身建设明显加强，民主法制和精神文明建设取得新成效。我们坚持依法行政、勤政为民，努力用务实的措施落实各项政策，政府执行力和公信力不断提高。

依法行政稳步推进。加快政府职能转变，法制监督得到加强，法治政府建设不断深入。认真执行人大及其常委会的决议，主动接受人大及其常委会的法律监督和工作监督，虚心接受人民政协和民主党派、工商联、无党派人士、人民团体的民主监督，累计办理市级人大代表建议857件、政协委员提案1479件。“五五”普法通过省级验收，社会矛盾纠纷“大调解”机制逐步完善，荣获“全国社会治安综合治理优秀市”称号。“四访促和谐”活动扎实开展，一批疑难信访问题得到化解，群众诉求渠道进一步畅通。安全生产形势持续稳定好转，生产安全事故四项指标全面下降，连续六年被评为全省安全生产责任目标考核优秀单位。

精神文明创建取得实效。注重用深厚的文化底蕴塑造城市精神，用身边的先进典型教育广大市民，开展“感动荆州”年度人物、“十佳小巷总理”、“荆州好人”等评选活动，涌现出“感动中国人物”荆州“10.24”英雄群体、“全国道德模范”赵传宇、“全国医德标兵”吴诗琦、“全国十大杰出检察官”马骏镠、“全国兴村富民百家领军人物”叶昌保、“刚正敬业的好干部”高荆洪、“见义勇为模范家庭”杨振环一家、“舍己救人英雄”刘飞和高维、“模范村党支部书记”刘仁富、“人民的好卫士”王锟和“6.27”英雄群体等模范人物，奥运圣火荆州传递活动成为市民文明素质的完美展示。

服务型政府建设得到加强。深入开展学习实践科学发展观和学习型机关、服务型政府、创新型城市创建活动，政府执行力建设和作风建设年、能力建设年活动扎实推进，科学发展水平、行政服务效率进一步提高。政风行风评议、绩效考核机制不断健全，行政问责、执法检查和审计监督不断加强，政务公开、政务督查、应急管理水平不断提高，政风建设、廉政建设取得新成效。坚持每年为群众办理10件实事，一批涉及民生的实际问题得到妥善解决。

六年成就辉煌，令人鼓舞自豪。特别是刚刚过去的2010年，全市经济社会持续快速协调发展，呈现阶梯式上升的良好态势。地区生产总值增长13.2%，增量超100亿元；财政收入增长46.7%，增量超20亿元；固定资产投资增长38.1%，增量超100亿元；农民人均纯收入增长18.1%，增量近1000元，均创历史新高，成为荆州快速发展的里程碑。面向未来，荆州发展已经站在一个新的历史起点上。

各位代表！我市经济社会发展成绩来之不易，这是省委、省政府和市委正确领导的结果，是全市上下解放思想、负重前行、开拓创新、团结奋斗的结果。在此，我代表市人民政府向为荆州改革发展稳定作出积极贡献的广大干部群众，向全市人大代表、政协委员、各民主党派、工商联、无党派人士和人民团体，向驻荆部队、武警官兵、政法干警和部省垂管单位，向所有关心支持荆州建设与发展的海内外朋友，表示衷心的感谢并致以崇高的敬意！

各位代表！六年的工作实践，我们得到了一些有益的经验与启示：必须坚持解放思想不停步，着力突破体制机制、发展要素、思路胆略等瓶颈，不断开创发展新局面；必须坚持“工业兴市”战略不动摇，突出工业主体地位，调整优化经济结构，转变经济发展方式；必须坚持统筹兼顾不偏颇，在推动经济快速发展的同时协调抓好社会事业，在加快新型工业化的同时扎实推进城镇化和农业现代化，在提升中心城区竞争力的同时注重增强县域经济实力；必须坚持勤政为民不懈怠，始终把保障和改善民生作为政府工作的出发点和落脚点，努力让人民群众在发展中得到更多实惠，形成凝心聚力的强大合力。

回顾过去的六年，我们也清醒地认识到，我市经济社会发展还存在一些亟待解决的矛盾和问题：经济总量仍然偏小，缺乏支撑和带动力强的大产业、大企业、大项目，战略性新兴产业尤显不足，加快转变发展方式的任务艰巨；体制机制障碍较多，城乡二元结构明显，一些重点领域和关键环节改革尚需突破；地方可用财力有限，城乡基础设施欠帐较多，农民持续增收难度加大，就业、住房、社会保障、社会治安、城市管理等方面与人民群众的期望还有较大差距，改善民生和维护稳定的任务繁重；少数部门办事效率不高，执行力不强，还存在一些消极腐败现象。面对这些问题，我们决不回避、决不退缩，积极采取措施，切实加以解决。

二、今后五年的发展展望

“十二五”时期，是我市全面建设小康社会的关键时期，也是我市全面发力、跨越发展的攻坚时期。在新一轮大发展中，我们面临的总体环境较好，有利条件较多。

从外部发展环境看：我国发展仍处于大有作为的战略机遇期，发展的内在优势和长期向好的趋势没有改变，经济正在由较快增长向稳定增长过渡；国家支持湖北建成促进中部崛起的重要战略支点，加强“后三峡”政策扶持，加快水利改革发展，支持粮食主产区、中国大遗址保护荆州片区建设，实施“十二五”老工业基地调整改造规划，面向我市的政策机遇正在汇集；省委、省政府实施“两圈一带”战略，支持我市发展综合交通，支持宜荆荆城市群、仙洪新农村试验区建设，将为我市跨越发展提供千载难逢的机遇。

从内部发展环境看：“十二五”时期，汉宜高速铁路、荆岳铁路、荆州组合港等一批重大基础设施项目建成投运，美的、锦辉、飞和等一批重大产业项目带动引领，丰富的水资源、农产品资源、文化旅游资源和卤水资源优势凸现，将为我市跨越发展提供强大支撑；历届党委、政府致力于“打基础、管长远”，经济快速增长的累积效应、“工业兴市”战略的释放效应和基础设施建设的联动效应逐步显现，将为我市跨越发展提供基础条件；全市广大干部群众思发展、谋发展、促发展的氛围日趋浓厚，将为我市跨越发展提供有力保证。

同时，我们也要看到，国际金融危机还没过去，甚至会出现反复；周边地区竞相发展，也将给我们带来巨大的竞争压力；我市在经济快速发展中一些长期积累的深层次矛盾和问题尚未彻底解决，今后发展还将面临许多新的困难和挑战。只要我们坚定信心，抢抓机遇，乘势而上，一定会实现荆州的跨越发展。

今后五年的指导思想是：坚持以邓小平理论和“三个代表”重要思想为指导，深入贯彻落实科学发展观，抢抓中部崛起、“两圈一带”战略机遇，以科学发展为主题，以加快转变经济发展方式为主线，以改革创新为动力，以推进新型工业化、新型城镇化和农业现代化为重点，着力构筑大交通，培育大产业，建设大城市，更加重视保障和改善民生，更加重视社会稳定，全面建设和谐荆州，推进荆州跨越发展，努力建设湖北长江经济带“钢腰”、江汉平原中心城市和长江沿岸经济重镇，为全面建成小康社会打下坚实基础。

今后五年的主要发展目标是：力争全市生产总值年均增长13%以上，规模以上工业增加值年均增长25%以上，全社会固定资产投资年均增长20%以上，社会消费品零售总额年均增长18%，外贸出口年均增长20%，地方一般预算收入年均增长13%以上，城镇居民人均可支配收入年均增长13%以上，农民人均纯收入年均增长13%以上，人口自然增长率控制在6‰以内，城镇登记失业率控制在4.5%以内，单位生产总值能耗下降16%。力争2015年地区生产总值、固定资产投资、地方一般预算收入分别在2010年基础上翻一番，年均增幅高于全省平均水平。

今后五年的主要工作思路和措施是：

（一）培育大产业，提升发展竞争力。把产业发展作为跨越发展的重中之重，培育以工业为主导、以农业为基础、以文化旅游为特色、以科技创新为驱动的大产业，努力建设经济强市。加快发展现代工业，实施“223”产业发展计划，改造提升传统产业，培育农产品深加工、化工及新能源两大千亿元产业，装备制造、轻工两个500亿元产业，汽车及零部件、纺织印染服装、电子信息三个200亿元产业；突破性发展战略性新兴产业，重点培育电子信息、生物医药、节能环保、新材料、新能源汽车、高端装备制造业，为未来发展赢得主动、争创优势。加快发展现代农业，着力发展水产、水稻、油菜、水禽产业，建设全省最大的优质稻生产基地、全国最大的双低油菜、淡水水产品生产基地；突破性发展农业产业化经营，构建粮棉油、瓜果菜、畜禽、水产、林纸等七大农产品加工产业体系；加快推进“四个一批”工程，打造6个过百亿元加工大县，建成10个产值过50亿元的加工园区，培植10个产值过50亿元的龙头企业和10个驰名商标，力争农业产业化水平进入全国前列；抢抓国家重视水利改革发展的机遇，加强以农田水利为重点的农业基础设施建设，下大力改善水生态、水环境。加快发展现代服务业，以鄂西生态文化旅游圈建设为契机，突破性发展文化旅游业，加快建设中国大遗址保护荆州片区、中心城区文化旅游板块、洪湖湿地生态和红色旅游板块、松滋洈水自然风景区旅游板块、石首天鹅洲湿地旅游板块，努力打造以楚文化、三国文化、水文化为特色的国内一流、世界知名的旅游目的地。同时，大力发展生产性和生活性服务业，建设长江中游重要的现代物流基地，努力打造鄂中南地区商贸中心城市。

（二）构筑大交通，提升发展辐射力。把交通先行作为跨越发展的基础条件，抢抓湖北长江经济带新一轮开放开发机遇，加强公路、铁路、港口、能源、信息等重大基础设施建设，努力建设以大路网、大港口、大物流、大管网为支撑的中部地区重要的综合交通枢纽。按照“三横五纵”的高速布局，构建内畅外联的公路网，规划建设洪监、江南等5条高速，江监、仙洪赤、荆松宜3条一级公路，荆州长江二桥等4座过江通道，实现县县通高速，打造“半小时经济圈”。构建通达全国、覆盖县市的铁路网，建成汉宜高铁荆州段、荆岳铁路，彻底改写荆州铁路运输落后的历史。充分利用长江黄金水道优势，按照“一港十区”的总体布局，打造荆州亿吨组合港，形成辐射“两湖”平原，水、公、铁无缝隙对接的综合枢纽港口群。整治荆江河段，建设引江济汉工程。加快推进松滋核电、江陵火电、国电长源二期等重点能源项目建设，探索开发地热资源、太阳能、生物质能、沼气等再生能源。积极推进电信、广播电视、计算机通信三网融合和“物联网”应用，努力建设“数字荆州”。

（三）建设大城市，提升发展承载力。把提升中心城区核心竞争力作为跨越发展的战略举措，坚持以规划为龙头，建设、管理、服务并重，充分展示历史文化名城魅力，积极争创国家卫生城市、国家园林城市、国家环保模范城市和省级文明城市，彰显滨江、生态、园林特色，营造宜居、宜旅、宜业、宜商的城市环境。按照“北越南跨、西进东联”的思路，拓展城市空间，拉开城市框架，力争市区城镇建成区面积达到100平方公里、非农业人口达到100万人。按照功能分区、分片推进的思路，积极推进荆州古城人口疏散和旧城改造，加快推进开发区中心商务区、沙北新区、火车站片区、海子湖生态文化旅游区、李埠临港新区建设，全面提升城市形象和功能。推动县域城市扩容提质，围绕“一城一港一区”，打造节点城市、综合港口、特色园区，将洪湖、监利、石首、公安、松滋建成城区人口过20万的中等城市。积极引导人口、产业向城镇集中，规划建设沿江、沿路城镇带，扶持重点镇、中心镇加快发展，打造一批功能完备、环境优美、特色鲜明的精品城镇，力争城镇化率达到50%。按照“以城带乡、城乡一体”的要求，加快新农村建设步伐，建设农民的幸福美好家园。

（四）推进大开放，提升发展内动力。把深化改革、扩大开放作为推动经济发展转型的先导战略，为实现跨越发展提供强大动力，努力建设湖北长江经济带开放开发的先行区、示范区。深化重点领域和关键环节改革，纵深推进国有企业和国有资产管理改革、农村综合配套改革，积极推进行政机构、行政审批、财政、金融、投资、医药卫生、文化、教育等领域改革，着力构建充满活力、富有效率、更加开放的体制机制。深入实施以“法制创新、行政提速、诚信荆州、优质服务”为主题的优化环境工程，努力把荆州建设成为中部地区最具投资价值的创业城市。建立健全区域创新体系，实施“质量兴市”战略、商标战略，培育精品名牌，加快建设创新型城市。全面加强开放平台建设，实施开发区升级工程，打造差异化竞争优势和产业集聚优势。重点支持荆州开发区争创国家级开发区和承接东部产业转移示范区，加快体制创新、机制创新、服务创新和基础设施建设步伐，努力打造成为全市改革开放的龙头、经济发展的引擎、企业家创业的乐园。坚持“引进来”、“走出去”，加强区域合作，强化招商引资，构筑对内对外开放新格局，建设开放型城市。

（五）促进大和谐，提升发展凝聚力。把保障和改善民生作为当前和今后的一项根本任务，加大对社会事业和公共服务的投入，着力实施创业就业、社保救助、教育均衡、生命健康、文化普惠、生态环保等行动计划，努力建设和谐荆州。大力推进创业带动就业，创建国家级创业型城市。健全覆盖城乡居民的社会保障体系，实现城乡统筹和应保尽保。完善城乡社会救助体系，发展社会福利和慈善事业。加强城镇保障性住房建设，逐步实现“住有所居”。大力推进科技创新，加快科技成果转化。以促进教育公平为重点，统筹发展教育事业，建设教育强市。以培养创新型人才为重点，认真组织实施《中长期人才发展规划纲要》，建设人才强市。以保基本、强基层、建机制为重点，加快发展医疗卫生事业，健全城乡医疗卫生服务体系。深入实施血防整县推进工程，力争达到血吸虫病传播控制标准。完善计划生育利益导向机制，稳定低生育水平。大力发展循环经济和低碳产业，推进重点行业、重点领域节能减排，走绿色发展之路。继续实施水安全保障、水资源开发、水环境整治和水文化建设四大工程，建设全国人水和谐示范区。深入开展中国特色社会主义核心价值观教育，推进文明创建，提高市民素质。认真办好第十四届省运会，加强非物质文化遗产传承，加大文化基础设施投入，大力发展文化事业和文化产业，建设文化荆州。坚持依法行政，全面推进“六五普法”，建设“法治荆州”。加强社会建设，创新社会管理，完善基层民主制度，加强社会治安综合治理，维护社会和谐稳定，积极争创省平安市。

三、努力实现“十二五”良好开局

2011年是实施“十二五”规划的开局之年，是推进跨越发展的关键之年，做好今年的工作意义重大。综合分析发展环境，立足荆州发展实际，建议今年经济社会发展的主要预期目标为：地区生产总值增长13%以上，规模以上工业增加值增长20%以上，全社会固定资产投资增长25%，社会消费品零售总额增长18%，地方一般预算收入增长13%，外贸出口增长15%，居民消费价格涨幅控制在4%左右，城镇居民人均可支配收入和农民人均纯收入分别增长12%和10%，城镇登记失业率控制在46%以内，人口自然增长率控制在6‰以内，单位生产总值能耗下降4%，化学需氧量下降1%，二氧化硫排放下降1%。

实现上述目标，要着力抓好以下八个方面工作：

（一）着力实施“工业兴市”战略，增强工业经济整体实力。坚持“工业兴市”战略不动摇，按照“低碳”、“生态”发展理念，加快产业转型、升级、集聚步伐，促进产业结构优化和质量效益提升，培育荆州工业发展新优势。

加快企业自主创新。把自主创新摆在工业转型的突出位置，充分发挥企业自主创新主体作用，鼓励企业加大核心技术的攻关力度，开发自主知识产权产品。加快荆州开发区国家级创业中心、省级以上企业工程中心和技术中心以及公共服务平台建设，开展产学研合作，推进科技成果向现实生产力转化。大力实施“质量振兴”工程，推进信息化与工业化的深度融合，推行企业卓越绩效管理模式，开展“荆江质量奖”评选活动，引导企业争创精品名牌。积极申报重大科技专项，运用先进适用技术改造提升传统产业，推动支柱产业向更高层次迈进。

加快园区产业集聚。深入实施“满园工程”，加强园区基础设施建设，强化园区招商和配套服务，促进要素资源向园区集聚。加快发展特色园区，中心城区重点抓好荆州开发区“六园两中心”、荆州区九阳工业园、沙市区银湖中小企业城建设，鼓励园区共建、差异化发展。抓住湖北长江经济带开放开发机遇，加快造船、仓储、物流等沿江产业园区规划建设。实施“抓大育小”工程，启动“223”产业发展计划，做强做优现有企业，强力招商引进一批企业，大力推进支柱产业发展，努力培育新兴产业。抓好融资担保、技术开发等配套服务，支持中小企业向专、精、特、新方向发展。

加快县域工业发展。用足用好省扶持县域经济发展的专项资金，从全民创业、园区建设、招商引资、软环境优化、企业家队伍建设入手，不断培育市场主体，发展壮大支柱产业，提高县域工业经济规模和实力，力争每个县市建成1-2个产销规模过50亿元的产业。强化县域工业量化考核，推动县域经济争先进位。

（二）着力发展现代农业，加快推进新农村建设。坚持城乡统筹，以农业增效、农民增收为核心，以推进农业产业化为突破口，促进农业、农村经济转型，努力提高农业综合生产水平、农业现代化水平和农民生活水平。

扎实推进农业结构调整。抓好以粮食为重点的粮棉油生产，认真组织实施新增粮食产能工程，确保粮食增产1亿斤以上，优质油菜产量稳定在1000万担以上，棉花总产稳定在260万担以上。坚持“重抓水产”战略，狠抓精养渔池改造升级，狠抓名特优水产品生产，力争全市放养面积达到220万亩，水产品产量达到110万吨。认真组织中国荆州淡水渔业博览会、“洪湖渔家”生态鱼香港推介会，着力抓好水产品进超市、进宾馆、进餐馆活动，进一步提升荆州水产在全国的影响力。稳步发展畜牧业，加快发展畜禽标准化、规模化养殖。加快农机化步伐，继续实施“以机代牛”整县推进工程，力争农机综合作业率达到60%。加快农业科技公益服务体系建设，推行“县级主管部门派出制”服务模式，推广轻简化栽培、测土配方施肥等实用技术，做好重大动物疫病、农作物病虫害防控工作，努力提高农业社会化服务能力。

扎实推进农业产业化经营。加快转变农业发展方式，坚持用现代物质条件装备农业，用现代科学技术改造农业，用现代产业体系提升农业，用现代经营形式推进农业，促进农业生产经营专业化、标准化、集约化。继续实施“四个一批”工程，重点加强农产品加工园区和龙头企业建设，突破性发展农产品加工业。支持和引导龙头企业采取兼并重组、资本联合、上市融资和品牌整合等多种形式，促进龙头企业进一步做大做强。加快发展农民专业合作社，完善利益联结机制，积极引导龙头企业和合作社建基地、联农户，提高农民组织化程度。加强农业精品名牌建设，健全农产品质量安全和农业投入品监管体系，提高农产品质量安全水平。

扎实推进新农村建设。完善新农村经济发展、基础设施、村庄布局等规划，防止低水平重复建设。抢抓中央1号文件聚焦水利建设的机遇，重点抓好荆江大堤综合整治、荆南四河堤防加固、四湖流域综合治理和水库除险、泵站更新改造等重大水利工程。继续抓好仙洪试验区荆州片区建设，努力建成全国新农村建设的样板；以点带面、全面推进，重点抓好省级试点镇、市级示范区、县级示范村建设，推进农村新社区和宜居村庄建设。推动城市基础设施和公共服务向农村延伸，加大农村路网电网、安全饮水、“一建三改”、危房改造投入力度，抓好清洁种植和清水生态养殖“两清工程”、农药和化肥减量化“两减工程”，全面提升新农村建设整体水平。大力支持党员群众服务中心建设，发展壮大村级集体经济。深入推进农村扶贫开发，切实增强老区贫困地区造血功能和发展能力。扎实做好防汛抗旱工作，提高农业防灾减灾能力。狠抓强农惠农政策落实，规范涉农收费，以“一折通”形式及时足额发放各项补贴资金，实施政策减负、政策富民。

（三）着力扩大内需，拉动经济持续快速增长。坚持投资消费双轮驱动，积极适应投资、消费方式转变的新形势，努力以项目带动投资，以消费促进增长。

扩大投资总量。发挥投资对扩大内需和调整结构的引导作用，加强项目策划、储备和申报工作，做到争取中省投资的份额不减、重点项目建设的力度不减、扩大民间投资的举措不减，不断提高投资规模、质量和效益。强化投资调度和协调服务，突出抓好锦辉富钾卤水开发、飞和汽车、美的电器、四机钻采、江陵美芳再生铝、公安中粮、松滋白云边酒业、监利福娃食品等一批投资过亿元的重大工业项目建设；加快荆州火车站、荆岳铁路、荆沙铁路改造、洪监高速、江南高速、318国道荆州段改线工程、引江济汉工程等一批重大交通项目建设；开工建设500千伏荆州南输变电工程，建成投产220千伏居正变电站。加强高成长性后备上市企业的培育和辅导，推动企业上市和债券发行，拓宽企业直接融资渠道。

扩大消费需求。进一步优化城市商业网点布局，推进中心城区核心商圈建设。加快城市商业综合体、小商品城等项目建设，培育商业新亮点。改造提升传统商贸服务业，运用现代经营方式促进商贸、餐饮、酒店等提档升级，加强生产资料和农产品产地批发市场建设，发展壮大两湖绿谷、蓝特建材、洪城商港等专业市场。积极发展现代物流业，扎实开展国家农产品现代流通综合试点，高起点规划建设荆州火车站物流园、荆州港保税物流园、荆襄物流配送中心等重点物流园区，积极推广连锁经营、网络配送、电子商务等消费方式。认真落实家电、汽车、摩托车、农机下乡等政策，推进“万村千乡市场工程”，发展村级综合服务社，不断激活农村消费。保持打击销售非法卷烟的高压态势，净化卷烟市场。

培育文化旅游支柱产业。围绕“楚国故都、三国名城”旅游发展主题，完善荆州古城核心景区、海子湖生态旅游新区等专项规划，编制县市区重点旅游项目规划。做实文化旅游投资平台，推动中心城区旅游资源整合和经营权转让，吸引国内外大企业、大集团参与旅游开发。加快推进纪南城遗址公园、关公文化园、熊家冢遗址博物馆、八岭山考古遗址公园、荆州博物馆新馆、市文保中心、“垄上行”文化产业园、“洪湖岸边是家乡”、洈水风景区等景点建设，打造荆州文化旅游品牌。实施文化产业培育工程，做大做强广播电视、新闻出版、文化娱乐、创意动漫、教育培训、体育休闲等文化产业，提升荆州文化品位。认真组织中国大遗址保护高峰论坛和旅游专场推介会，精心策划关公祭朝觐等活动，让更多荆州文化元素走向全国，走向世界。以汉宜高铁通车为契机，加强城市合作，开拓客源市场，力争接待游客人数、旅游收入增长30%以上。

（四）着力完善城市功能，加快推进新型城镇化。坚持以“四城同创”为抓手，在更高起点上推进城市基础设施、环境质量和风貌特色建设，努力提高市民的幸福感和满意度。

提高城乡规划管理水平。抓好新一轮荆州城市总规的宣传展示，抓紧完善城乡规划体系，提高城镇控规覆盖率。健全规划实施监管体系，理顺城乡规划管理事权，加大执法力度，维护规划的权威性和统一性。

加快城市基础设施建设。中心城区按照“新区开发、老城提升、组团展开、整体推进”的思路，加快完善以道路交通、城市管网、园林绿化、垃圾处理等为重点的基础设施建设。加强城投公司融资平台建设，提升经营城市水平。建成荆楚大道，改造屈原南路、凤凰路、荆秘路、锅底渊路、三湾路等城市主干道，抓好小街巷、城市出入口改造工程，建设荆襄外河湿地公园、雄楚公园、新植物园、范家渊公园，启动中心城区沿江大堤综合整治，加快推进水、电、气、热等市政基础设施建设，满足生产生活需求。整治西干渠、豉湖渠等内河水系，疏通中心城区排水通道。抓好规划见绿、建景显绿、拆违还绿、拆墙透绿，建设一批城市生态林、道路景观、园林社区，真正让城市绿起来、美起来、亮起来。加大棚户区、城中村改造和经济适用房、公共租赁房建设力度，增加中低价位、中小户型商品房供给。继续实施“百镇千村”示范工程，引导农村人口向城镇集中、工业企业向园区集中、生活居住向小区集中。

推进城市精细化管理。坚持“建管并重、重在管理”，深化城市管理体制改革，推进绿化、环卫、市政等管养分离、市场化运作、社会化管理，强化区级职能，落实管理责任，实行城管处罚权相对集中。推进城市管理网格化，健全精细化、常态化管理机制。继续抓好社区环境、交通秩序、集贸市场和早夜市摊点等十大专项整治，解决好乱搭乱建、占道经营、交通拥堵等顽疾，努力实现创建国家园林城市、省级文明城市目标。

（五）着力推进改革开放，不断增强经济社会发展活力。坚持解放思想，用创新的思维、开放的理念、市场的办法破解发展难题，努力提高经济社会发展的可持续性。

进一步深化改革。抓紧完成国企改制煞尾任务，认真解决国有企业改制遗留问题。强化国有资产管理，引导企业建立现代企业制度，提高国有资产保值增值能力。巩固和深化农村综合配套改革，完善“以钱养事”机制，推进土地适度规模经营和承包经营权流转。继续推进行政审批制度改革，切实做到“三集中、三到位”。积极推进事业单位分类改革，稳步实施绩效工资制度。理顺教育管理体制，抓好市属高校、中职学校资源整合，支持民办教育发展。稳步推进医药卫生体制改革，办好基本药物制度和公立医院改革试点，建立四位一体、覆盖城乡的基本医药保障制度和医疗卫生保障体系。

进一步扩大开放。突出大招商、招大商，强化招商引资目标考核，力争全年到位资金300亿元，增长15%以上。举全市之力，加大简政放权、财政倾斜、基础设施投入力度，支持荆州开发区率先跨越发展，实现体制创新、政策改版、发展升级。创新招商方式，实现由招商引资向招商选资转变，让有限的环境资源发挥最大的招商效益；实现由单个项目招商向产业链招商转变，促进产业配套聚集；实现由传统的活动、会议招商向“一对一”式的跟进招商转变，提高招商引资的针对性和实效性，重点吸引世界500强、国内500强和公司总部、研发机构落户荆州。全力抓好征地、房屋征收、要素配套、周边环境整治等服务，认真解决签约项目落地难问题。重奖招商中介有功人员，探索专业招商人员绩效年薪制。鼓励企业“走出去”，利用国际国内两个市场、两种资源，保持外贸出口稳步回升势头。加强“大通关”服务和电子口岸建设，争取盐卡港通过国家一类口岸验收。

（六）着力抓好财税金融工作，提高发展保障能力。坚持科学聚财与规范理财并重，扩大信贷与防范风险并重，努力为经济平稳较快增长提供有力保障。

提高财税管理水平。积极培植财源，强化税收和非税收入征管，挖掘增收潜力，严格依法治税，做大财政蛋糕，增强市级财政调控能力。调整优化支出结构，从严控制一般性支出，重点保障“工业兴市”、科技创新、城市建设、社会事业、改善民生等方面的投入，集中财力办大事、办实事、办成事。进一步完善部门预算、国库集中支付、行政事业单位资产管理等制度，建立健全预算绩效管理和财政投资评审制度。加强财政科学化、精细化管理，切实提高财政资金使用效益。加强政府债务管理，防范和化解债务风险。

提高金融服务水平。进一步加强政银企合作，建立中小企业融资网上俱乐部。积极引导金融机构创新金融产品，优化信贷结构，加大对新型工业化、农业农村、中小企业和县域经济发展的支持力度。继续实施中小企业“牵手培植”和“信贷回流”工程，力争贷款余额新增120亿元，贷存比提高3个百分点，确保今年贷款增幅高于往年，高于全省平均增幅，中小企业贷款高于各项贷款的平均增幅。深入开展信用创建活动，建立金融、司法联席会议制度和案件执行协调机制，严厉打击失信违法行为，营造诚信、安全的金融生态环境。鼓励支持金融机构改革与发展，加快引进村镇银行和股份制银行，积极推动市商业银行战略重组和县级信用社改制，做大做强小额贷款公司和中小企业担保公司，提高金融服务经济的能力。

（七）着力保障和改善民生，协调发展各项社会事业。坚持公共资源配置更多地向民生倾斜，完善服务体系，创新服务机制，提高服务质量，逐步推进基本公共服务均等化，让发展成果惠及全市人民。

加强就业和社会保障。以创建国家级创业型城市为目标，以创业带动就业，多渠道开发就业岗位，加强职业技能培训，加强创业基地建设，统筹做好大中专院校毕业生、农村转移劳动力、城镇就业困难人员和退役军人等群体就业工作。加快建立覆盖城乡居民的社会保障体系，办好新型农村社会养老保险试点，推进城镇企业职工养老保险省级统筹和医疗、失业、工伤、生育保险市级统筹，力争社保参保人数达到370万人次，征收社保费26亿元。进一步提高保障水平，落实企业退休人员基本养老金调标、医疗保险提高待遇和新农合提标工作。健全完善社会救助体系，做好城乡低保、特困户救助、灾民救济等工作。

加快发展社会事业。坚持教育优先，调整教育结构，优化学校布局，支持在荆高校发展，下大力解决中小学乱收费、学生负担过重等问题。加大科技投入，加快科技创新体系建设，引导科技资源高效配置、集成集聚。以高层次科技人才、高素质企业家人才培养为重点，统筹推进各类人才队伍建设。提高医疗卫生水平，降低药品价格，让老百姓看病更省钱、更方便、更放心。加强公共卫生防疫，抓好结核病、血吸虫病、艾滋病等疾病防控工作。综合治理出生人口性别比，提高出生人口素质。深入开展全民健身活动，启动第十四届省运会筹备工作。实施文化惠民工程，加强文物保护和非物质文化遗产传承，发展广播电视、新闻出版、文学艺术和社会科学事业，促进文化事业繁荣发展。认真做好国防动员、民兵预备役、人民防空工作，扎实开展双拥共建活动，积极争创“全国双拥模范城”。做好编制、外事侨务、台湾事务、民族宗教、移民、盐务、邮政、供销、气象、地震、档案、史志、保密和无线电等工作，重视和支持老龄、残疾人事业发展。狠抓节能减排，抓好工业、生活污染和农村面源污染防控，强化污染防治设施运行监管，实施湿地生态修复，保护生态环境。加强基本农田保护，促进土地节约集约利用。深入开展群众性精神文明创建活动，提高市民素质。

保持物价基本稳定。做好价格舆论引导，稳定社会预期。加强市场价格监管，严肃查处恶意炒作、哄抬物价等不法行为。严格执行价格补贴政策，建立社会救助、保障标准与物价上涨挂钩的联动机制。进一步扩大住房公积金制度覆盖面，提高住房公积金使用率。落实国家房地产调控政策，有效遏制房价过快上涨。

（八）着力创新社会管理，促进社会和谐稳定。坚持把维护社会和谐稳定作为发展之需、为政之责，增强工作的敏锐性、预见性、主动性和创造性，努力营造安定有序、共建共享的社会环境。

夯实社会维稳基础。加强城乡社区建设，扩大农村社区建设试点范围，推进社区建设全覆盖。创新社区管理和服务，支持民间组织和中介组织发展，推广沙市区“居民说事”经验，提高社区自治和服务功能。开展和谐社区创建活动，提升社会管理水平，努力把城乡社区建设成为管理民主、服务完善、文明祥和的社会生活共同体。

加强社会治安综合治理。坚持黑恶必除、命案必破，严厉打击涉黑涉恶涉毒、“两抢一盗”等违法犯罪行为，提高人民群众安全感和满意度。加强社会矛盾化解，完善人民调解、行政调解、司法调解和专业性仲裁调解相互衔接的“大调解”体系，继续加强行业组织和重点领域的第三方调解机制建设。健全重大决策社会稳定风险评估机制，从源头上预防和减少矛盾。继续抓好“四访促和谐”活动，广辟渠道，畅通言路，落实责任，化解矛盾，切实把群众的情绪疏导好、诉求解决好、利益维护好，努力实现“小事不出村、大事不出乡、难事不出县、矛盾不上交”。

抓好社会安全管理。强化食品、药品专项整治，严厉打击制假售假行为，确保食品、药品安全。全面落实安全生产主体责任，抓好生产安全、消防安全等专项整治行动，杜绝重特大事故发生。完善应急管理体制，加强综合应急救援队伍和平台建设，强化演练和预警预测，提高应急处置能力。

四、加强政府自身建设

坚持用亲民的理念维护民利，用创新的方法破解难题，用务实的举措落实工作，用廉洁的作风提升形象，不断增强政府凝聚力、执行力和公信力，努力建设服务型政府。

（一）强化依法行政。深入贯彻国务院《全面推进依法行政实施纲要》，不断规范行政行为，加快建设法治政府。自觉接受人大及其常委会的法律监督、工作监督，接受人民政协的民主监督，接受司法监督、群众监督、舆论监督，认真办理人大代表建议和政协委员提案。支持各民主党派、工商联及无党派人士建言献策、参政议政，发挥工会、共青团、妇联等人民团体桥梁纽带作用。推进电子政务和政府网站建设，完善新闻发布制度，加强“市长热线”建设，提高政府工作的透明度和公众参与度。健全重大事项集体决策、专家咨询、社会公示与听证制度，促进决策科学化、民主化、法制化。

（二）强化服务发展。坚持“产业第一、企业优先”，全过程、全方位、全天候为企业提供“保姆式”服务。加强学习，注重调研，推行“一线工作法”，提高为企业和群众解决实际问题的能力。倡导敢于担当、敢于碰硬、敢于求真的工作作风，对定下来的事情雷厉风行，务求落实。创新工作方式方法，突破体制、机制、资金等瓶颈制约，跳出“不敢想”、“不可能”的思维局限，努力开创各项工作新局面。继续开展创建“满意机关、满意站所、满意科长”活动，完善绩效考评办法，让人民群众拥有更多的发言权和监督权。优化审批流程，以“减程序、减时限、减费用”为重点，营造“低成本、零障碍、高效率”的行政服务环境，下大力解决办事难、办事慢等问题，努力使政策落实，项目落地，民生落户。

（三）强化从严治政。加强公务员队伍教育管理，强化宗旨意识、大局观念。严格追究行政机关失察、失管、失职行为，加大行政问责力度，坚决杜绝不作为、乱作为。严禁向企业伸手拉赞助、搞摊派，切实减轻企业负担；严禁向企业指定中介服务机构，解决中介组织与行政部门“明脱暗挂”问题；严禁粗暴执法、野蛮执法，推进柔性执法、温暖执法。充分发挥监察、审计职能作用，推进行政权力阳光运行，防止权力利益化、集团化。认真落实廉政建设“一岗双责”制，抓好纠风治乱工作。进一步规范土地出让、产权交易、招投标、政府采购、公务消费等行为，重拳治理工程建设领域突出问题，曝光典型案件，严查违法行为，维护清正廉洁的政府形象。

各位代表！我们将按照“促进公平、扶贫济困、尽力而为、当年完成”的原则，继续从群众最关心、最直接、最现实的问题入手，集中力量办好一批利民惠民的实事。一是新增城镇就业7万人，组织农村富余劳动力转移就业4.5万人，培养高技能人才5000人；二是中心城区筹集廉租住房400套，发放廉租住房租赁补贴8000户，启动建设公共租赁住房200套，实施城市棚户区改造20万平方米；三是继续开展新型农村社会养老保险试点，参保人数达到60万人，免费救治晚血病人2580人；四是为2.8万名高中贫困学生和5.8万名中职学生提供助学金；五是统筹推进新一轮“菜篮子”工程建设，新建蔬菜生产基地5万亩，新增设施蔬菜面积5000亩；六是提高农村低保对象补助水平，提高农村五保供养对象财政补助标准；七是新建农村候车亭188个，改造乡镇客运渡船50艘；八是建设农家书屋400家，为中心城区2个街道文化中心、40个社区文化活动室添置文化设备；九是免费为100名贫困下肢残疾人安装假肢，为200名贫困白内障患者实施复明手术，为1000名贫困精神残疾人提供基本控制药物，为200名6岁以下贫困残疾儿童实施抢救性康复，为困难群众和农民工办理法律援助案件1300件以上；十是对中心城区22个社区环境进行综合整治。

各位代表！实现跨越发展是荆州的必然选择，是全市人民的殷切期盼。让我们在省委、省政府和市委的正确领导下，在市人大、市政协的监督支持下，开拓创新，励精图治，求真务实，扎实工作，为圆满完成本次会议确定的各项目标任务，全面实现“十二五”宏伟蓝图而努力奋斗！
